# Asymmetries of Poverty: Why Global Burden of Disease Valuations Underestimate the Burden of Neglected Tropical Diseases

**DOI:** 10.1371/journal.pntd.0000209

**Published:** 2008-03-26

**Authors:** Charles H. King, Anne-Marie Bertino

**Affiliations:** Center for Global Health and Diseases, Case Western Reserve University School of Medicine, Cleveland, Ohio, United States of America; London School of Hygiene & Tropical Medicine, United Kingdom

## Abstract

The disability-adjusted life year (DALY) initially appeared attractive as a health metric in the Global Burden of Disease (GBD) program, as it purports to be a comprehensive health assessment that encompassed premature mortality, morbidity, impairment, and disability. It was originally thought that the DALY would be useful in policy settings, reflecting normative valuations as a standardized unit of ill health. However, the design of the DALY and its use in policy estimates contain inherent flaws that result in systematic undervaluation of the importance of chronic diseases, such as many of the neglected tropical diseases (NTDs), in world health. The conceptual design of the DALY comes out of a perspective largely focused on the individual risk rather than the ecology of disease, thus failing to acknowledge the implications of context on the burden of disease for the poor. It is nonrepresentative of the impact of poverty on disability, which results in the significant underestimation of disability weights for chronic diseases such as the NTDs. Finally, the application of the DALY in policy estimates does not account for the nonlinear effects of poverty in the cost-utility analysis of disease control, effectively discounting the utility of comprehensively treating NTDs. The present DALY framework needs to be substantially revised if the GBD is to become a valid and useful system for determining health priorities.

## Introduction

*“What cannot be counted simply doesn't count, and so we systematically ignore large and important areas of concern.”* — Ida Hoos, 1979 [1]. This statement is particularly relevant to assessing the impact of neglected tropical diseases (NTDs) on the world's burden of disability. Last year's Disease Control Priorities Project asked “How much health will a million dollars buy?” [2]. Because of flaws in the disability-adjusted life year (DALY) system that we use for counting up disease burdens, the answer must be “We really don't know.”

Two recent reviews [3],[4] and a linked viewpoint article [5] published in *PLoS Neglected Tropical Diseases* have introduced readers to the controversies surrounding the merits and demerits of the World Health Organization (WHO)–World Bank's 1996 Global Burden of Disease (GBD) program (Box 1) [6], as well as its current plans for its revision as GBD 2005 under the auspices of the Bill and Melinda Gates Foundation [3]. The problem of inaccurate disease-burden assessments is particularly acute for the diseases now characterized as neglected—in short, the NTDs [7],[8]. NTDs are chronic infections, including all helminthiases and many protozoal, bacterial, and fungal infections that are common among disadvantaged populations who live in less-developed nations. By contrast, NTDs are quite rare in the more affluent countries of the developed world.

Why are the NTDs “neglected” diseases? The question stems in large part from the use of the DALY (Box 2) as a health metric in policy planning, and the inadequate job done by the ongoing GBD programs in capturing the health and economic burden caused by NTDs in less-developed countries.

**Box 1. The GBD**

**Rationale for the Global Burden of Disease initiative ∼1991**

- Societies need to make decisions about their provision of health services.
- Policy makers must be aware of comparative disease burdens and the injuries and the risk factors that cause them.
- We need to understand the impact that modifying risk factors can have on global disease burden.
- Consensus must be reached about how to quantify the “importance” of risk factors in a way that is comparable across nations

From Lopez et al. Global Burden of Disease and Risk Factors, 2006 [69].

**Box 2. The DALY**

**DALY = Disability-Adjusted Life Year**

- A time-based measurement unit (metric) for estimation the health burden caused by different diseases
- Meant to be interchangeable and equivalent across all locations and cultures
- The “Like-as-Like” philosophy

**Background**

- Newly invented in the 1990s
- Developed by the Global Burden of Disease (GBD) Program [9]
- Initially funded by the WHO and the World Bank
- Current revisions facilitated by funding from the Bill and Melinda Gates Foundation

**Formula: the DALY = YLLs+YLDs**

- The DALY is a composite metric calculated from the sum of **Y**ears of **L**ife **L**ost (**YLLs**) and **Y**ears **L**ived with **D**isability (**YLD**s) for any disease

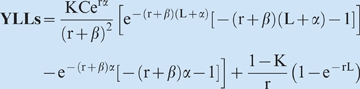

α = is the age at death

K = age-weighting modulation factor (K = 1)

C = is a constant (0.1658)

r = discount rate (0.03)

L = is the standard expectation of the life at age *a*

β = parameter from the age weighting function (b = 0.04)

- **YLL Simplified = (standard life expectation−age at death)×(age weight)×(future discount)**

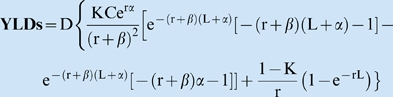

D = disability weight

L = duration of disability

α = age of onset of the disability

r = the discount rate (0.03)

β = the age-weighting parameter (b = 0.04)

C = adjustment constant (0.1658)

K = the age-weighting modulation factor (K = 1)

- **YLDs Simplified = (duration of disease)×(prevalence)×(disability weight)×(age weight)×(future discount)**

## Brief History

The heart of the GBD assessments, which were developed in the 1980s and early 1990s as a health-sector priorities collaboration between the WHO and the World Bank [3],[6], was the use of a new construct, the DALY (Box 2). The aim in developing the DALY was to objectively quantify and compare the aggregate regional and worldwide health burdens created by many different disease states.

One purpose of the DALY formulation was to create a scalable measure of disease impact for all health states, whereby an average disease impact per person could be assessed for any individual condition. Then, by knowing the total number of affected persons and the duration of the disease, the global “burden of disease” could be summed for that condition. This DALY approach and its ranking tables were believed to provide a more fair comparison of disease burdens, because the approach to ranking of diseases was believed to be nonsubjective, reflecting societal consensus, and avoiding the potential biases that had been involved in expert assessments of individual diseases [9]. The GBD program's ultimate agenda was to identify and rank preventable causes of injury and disease, which in turn was expected to lead to more effective implementation of strategies for disease control and prevention [10]. As we shall see, this goal was only partially met.

## DALY Drawbacks

The thesis of our current critique is that the design and use of the DALY involve inherent flaws that result in a systematic undervaluation of NTDs in world health. As we see it, there are three serious problems with the DALY system as it is used to rank the burden of disease caused by NTDs, as follows:

- A. By intentionally avoiding the “patient perspective,” the DALY system excluded local context as a modifier of disease impact.
- B. Many of the most common chronic complications of NTDs were overlooked as part of the evaluation and weighting of NTD-associated disabilities.
- C. In an effort to avoid overcounting actual life-years, the DALY scoring system did not address the reality of shared disabilities in the presence of comorbidities or concurrent infections.

In addition, despite the laudable intent of the original GBD program, serious criticisms of the DALY framework have come from many other sectors, including objections based on philosophical and ethical concerns about its approach to quantifying and discounting the value of disabled life [11]–[18]. Yet, despite these many cogent criticisms, no truly substantive changes have been made to the DALY system, and the use (or abuse) of the DALY metric has continued virtually unchanged since its introduction in the 1990s. A revision of the GBD (GBD 2005 [3],[4]) is in progress, and it is appropriate to re-address these issues in detail.

The DALY framers' initial intention was not that the DALY valuation should serve as a “norm” [9], yet the DALY has become normative because many health policy-makers and their funding partners use the DALY as their only measure of disease impact in programmatic analyses [11]. In essence, although most DALY users are not familiar with how a DALY is calculated, the DALY has become the primary value used to prioritize international investments in disease control [2].

To use a home-based analogy, DALY valuations determine whether it is a *luxury* or a *necessity* to spend available monies to treat human NTDs. If the DALY estimates are wrong, then the unfortunate consequence is that policy-makers' decisions about funding research or treatment programs for NTDs will undoubtedly be wrong as well.

## Some Specifics—The DALY Calculation

At heart, the DALY is a mathematical construct that models the health impact of individual diseases and allows them to be compared in various rankings (e.g., health economists' league tables). As with all such constructs, a number of simplifying choices and assumptions went into the creation of the DALY [18]. Not all of these assumptions were made explicit in the initial adoption of the DALY system (Box 3). Because they do not fully mirror reality, mathematical models, like roadmaps, are always caricatures, and in some senses wrong [19]. Despite their deficiencies, some models can be useful in our attempts to address specific complex questions, and the development of the DALY was thought to be a breakthrough [10].

Is the DALY truly useful in prioritizing health programs? From the NTD perspective, there is a suite of problems concerning many dimensions of the DALY that restrict its usefulness for making economic decisions for NTD control.

**Box 3. Hidden Assumptions of the DALY Approach**

1. There is an “average” disability for each disease state that is the same in all settings [9] **.**
2. There is a linear association between resource investment in a control program and the improvement of disease burden [10].
3. We assume that the “health consumer” is well informed and behaves rationally in making choices [47].

## Controversial Choices That Went into the Creation of the DALY

### The disability weighting scheme for the burden of nonlethal conditions

Most NTDs fall into the nonlethal category. That is, they are health states caused by communicable diseases in which impairment occurs because of long-term, disease-related inflammation. These infections only rarely cause sufficiently severe morbidity that they result in premature death [20].

The DALY calculation for nonlethal conditions is based on a years-lost-to-disability (YLD) calculation, using a disability weight (DW) that is intended to reflect the relative impact due to ill health from that particular disease during the period it afflicts an individual patient [21]. With time- and age-discounting adjustments, the DALY burden for a given disease reflects the sum of the number of people with that disease, multiplied by the time spent in that disease state, multiplied by the DW value (Box 2). Calculation of an accurate DALY value implies that one can quantify an accurate measure of disease impact (i.e., DW), as well as an accurate estimate of disease incidence and duration, or of current prevalence.

**Figure 1:**
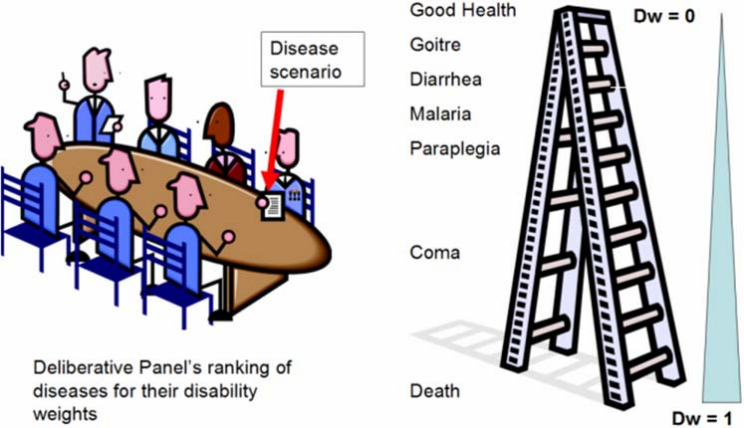
The DALY Person-Trade-Off Method of Disability Weight Determination.

DWs for the DALY system were determined by panels of nonexpert, highly educated participants assembled during the initial GBD programs of the late 1980s and early 1990s [9]. Their DW scores were assigned using an established (and, some might argue, nonintuitive [22]) health economics technique called the “person trade-off” (PTO) method (Box 4). The group's choices were benchmarked against a ladder of 22 indicator conditions that had been previously ranked by the same group or by other nonexpert groups (Figure 1). In assigning DWs, capsule scenarios of each disease state were provided to the PTO groups in order to guide their discussions. Where the group's choices disagreed, particularly between the two PTO methods, a group leader or facilitator required them to come to a consensus score for each condition. The resulting single point estimate for the DW is expressed on a scale from 0 to 1, where 0 denotes perfect health and 1 is death [9].

We posit that, while the PTO methods perhaps fits well with established economic theory about consumer preferences, from the health practitioner's and patient's perspectives, the PTO approach appears exceptionally awkward and unrealistic [22]. In the first series of questions (known as the PTO1 protocol), participants are asked to trade years of life of healthy individuals for the life extension of individuals in different disease states, i.e., “1 year of life for 1000 healthy individuals, or 1 year of life for 2000 blind individuals” [9]. In the subsequent PTO2 protocol, participants are asked to trade years of life extension in healthy individuals for years of life after morbidity alleviation (cure) for *N* individuals in a given disabling health state. Through additional group leader questioning and forced consensus of the DW-scoring groups, the answers from the two protocols are reconciled, and are then held to reflect the impact of this disease under “average social conditions” for the world at large [9].

For the GBD framers, the DW derived in this fashion is believed to reflect a “filtered consensus” of societal views about the impact of individual disease states [9]. Although the PTO is an apparently systematic and “value-free” approach grounded in economic practice, the assignment of DWs is, in fact, largely subjective. Who were the scoring panels, and how were they constituted and assembled? Who were the facilitators? Ustun and colleagues [23] have since systematically re-examined morbidity rankings for 17 conditions in 14 different countries, and noted significant differences between countries in their rankings for 13/17 conditions, with the most pronounced differences for the most stigmatizing illness, HIV/AIDS.

The details of GBD DW estimation have not been published in the peer-reviewed literature, although GBD literature [9] indicates that, because of the difficult nature of the PTO exercise, the panels had to be composed of highly educated individuals. “Training” to use the PTO and the enforced consensus among groups undoubtedly means that the perspectives of the group leaders and DALY designers were consciously or unconsciously imposed upon the DW ranking system. We see a strong possibility that the “filtered consensus” in fact reflected the individual and cultural biases of the panels and of the GBD facilitators themselves [11],[18],[24]. Without additional validation, published DW values have undoubtedly enshrined prevalent prejudices (or the frequently misinformed “common knowledge”) about individual disease states [17]. Despite this potential threat to the validity of the GBD valuations, the DWs have not been revisited or revised since their initial publication in 1996.

Can there be a single, “average” DW for a given health condition that is suitable worldwide for health burden calculations? Where significant population stratification exists, and risk for disease varies strongly among these strata, good statistical practice says that *there can be no valid global average* [25]. For NTDs, socioeconomic status (SES) modifies or confounds the lifetime risk of acquiring infection, meaning that poor communities jointly suffer more from disease-associated health burden. At the same time, other aspects of poverty serve as “effect modifiers” that worsen the impact of infection and restrict access to care. Where such significant stratum-related differences exist, the appropriate approach is to provide stratum-specific DWs, or create a weighting system that adjusts the DW for local SES. While it is not technically feasible to have a separate DW for every disease in every location [13], it would be highly appropriate in the next iteration of GBD (GBD 2005 [3]) to use local levels of poverty as an adjustor for disease DW and in its estimates of local, regional, and national disability impact. Like others, we believe that evidence-based adjustment of DWs for disease context [23] will be an essential step in creating more accurate GBD valuations [23],[26].

There is concern that revision of one or only a few DWs would unbalance the GBD rankings and possibly exceptionalize or overvalue selected diseases. In a WHO-requested re-review of the burden of disease due to schistosomiasis, the evaluators' conclusion was that “…it is unlikely that more accurate estimates would significantly change the ranking of schistosomiasis burden…” [27]. The implication was that revision of the GBD league tables was not required. Yet, it is important to see that in a health economics and policy planning environment where “cost per DALY averted” [2] drives many health policy decisions, having an accurate DW assessment would be an essential tool for making valid cost-related and policy judgments. Failure to adjust GBD calculations for location will result in a continued overvaluation of the “importance” of noncommunicable diseases (the primary disease burden of wealthier countries) with a significant undervaluation of the significance of communicable diseases (including the NTDs) that remain a dominant health burden for developing countries [18]. The inherent bias of the original GBD in favor of noncommunicable diseases has been carried into the current Disease Control Priorities Project [28], resulting in serious undervaluation of the importance of communicable disease control in current policy discussions.

**Box 4. Example of the Person Trade-off Process—PTO Exercise 1**

- You are a decision maker who has enough money to buy only **one** of **two** mutually exclusive health interventions:
-
  1. Intervention A or Intervention B.
- **Intervention A** will extend the life of **1,000 healthy (non-disabled) individuals** for **exactly one year.**
-
  - If you purchase intervention A, you will extend their lives for one year, at which point they will all die.
  - If you do not purchase intervention A, they will all die today.
- **Intervention B** will extend the life of ***N*** **disabled individuals** for **exactly** **1 year**; if you do purchase intervention B, they will die at the end of one year.
-
  - The alternative use of your scarce resources is Intervention B; if you do buy Intervention B, your disabled individuals will live for one year, at which point they will all die.
  - If you do not buy intervention B, they will all die today.
- **What is your value for** ***N?***

See [9] for details.

### Confusion about what is included in the diagnosis of an NTD

As Mathers et al. put forward in their recent review [3], “…the impact of highly prevalent diseases with smaller levels of morbidity has not been well measured [in the GBD system]…”. Hence, DWs due to NTDs created by the PTO process are problematic because, upon reflection, the scenarios used to determine disability in NTDs must not have reflected the full health impact of these conditions. The published DWs for the NTDs suggest to us that their scenarios were based on a limited understanding of the NTD-associated health states, or on an artificially restricted definition of these disease entities.

Where NTDs are prevalent, the bulk of their disease burden is in the form of low-level, chronic morbidity. While this situation might seem negligible to someone without experience of this group of diseases, it must be remembered that most NTDs persist for years (at least half a lifetime) and continue to affect personal health and performance status for decades, even after infection is cleared. Health evaluations in certain areas of Japan, China, and North Africa where schistosomiasis transmission has ended indicate there is a significant long-term disease impact of “post-transmission schistosomiasis” [29] that must be addressed in health planning. In view of the large numbers of people who carry NTDs and the long duration of NTD effects, the aggregate years of healthy life lost to chronic NTDs must be large. Yet the DALY values presented for most NTDs in the GBD tables do not reflect this reality. Why should this be so?

NTDs often present in the context of multiple coinfections. Polyparasitism is a fact of life where most NTDs occur [30]–[32]. As such, it has been difficult to disaggregate the “attributable risk” belonging to individual pathogens when we consider the causation of infection-associated morbidities [33]. Where complications such as anemia or malnutrition were recognized, inherent problems in determining cause-specific attributable risk led to these health- and performance-significant outcomes being disaggregated from the PTO scenarios and therefore from the assigned DWs of many of the NTDs [34]. However, if properly valued and included in DALY calculations, these more “subtle” morbidities could, in fact, make up the bulk of prevalent morbidity and disability for many chronic NTDs.

Part of the problem with the available data on NTD-related morbidity is due to significant limitations in the design of past population-based surveys [26]. Where resources are limited, study sample size tends to be small. Large clinical effects and clinical outcomes unique to individual diseases were easy to measure in small surveys [35]. However, more “subtle” pathologies, and those having mixed etiology were much harder to measure precisely, and often when they were found to be “not statistically significant” they were ignored (in a classic type II error) or dismissed as clinically unimportant 36,37.

We posit that the GBD specifically did not want to overcount DALYs. That is, in their overall schema, there could not be more life-years and DALYs reported as lost than there were life-years lived by the global population. This “no overcount” rule appears to have led the framers to disaggregate important comorbidities such as anemia, diarrhea, growth stunting, and cognitive impairment from many of the disease outcome assessments [34]. However, it can be seen as fundamental flaw of the DALY system that its “disease” categories are based on a classification system that includes both etiologic disorders and undifferentiated syndromes (e.g., “anemia” and “infertility”) as separate disease entities. The system that the GBD used, the International Classification of Diseases, ninth edition (ICD-9), is a convenient off-the-shelf classification system for reporting health statistics, but it is seriously deficient in defining preventable causes of disease, as the GBD originally set out to do.

In medical practice, it is ethically unacceptable to leave a patient with only a syndromic diagnosis such as “anemia” or “growth stunting,” without making a concerted effort to establish the underlying etiologic (causal) diagnosis. In like fashion, it is inappropriate for the DALY system to disaggregate NTDs from their common infection-associated morbidities, including the syndromes of anemia, growth stunting, and cognitive impairment. In disaggregating these morbid complications from their infectious causes, the DWs assigned to specific NTDs are thus mistakenly cheapened. When NTDs are viewed merely in this limited fashion, they appear “unimportant” when compared to more acute or more lethal disorders. It is noteworthy that the GBD insisted on redistributing any deaths reported as due to “[s]ymptoms, signs and ill-defined conditions,” reclassifying them into known ICD groupings [34], yet it failed to determine “attributable burden” for disability due to most nonlethal syndromic conditions [38]. This devaluation, entrenched by the GBD “…because we were unable to locate sufficient evidence on the relative risk…” [38] is then (as part of the DALY scores) imputed to reflect societal preference [9] and, as such, functionally enshrines the “neglect” of the NTDs in health policy.

It is well known that anemia and the other GBD-listed syndromes have many distinct preventable causes, including the NTDs [39],[40], which need to be identified, clearly defined, and appropriately treated with specific remedies. We suggest that every effort should be made to estimate the pathogen-specific attributable fraction [41] of these undifferentiated syndromes in order to appropriately reassign their disability burden to specific pathogens, which, in turn, represent the truly preventable disease etiologies. Certainly, improved diagnostics may be needed to more accurately determine who is infected with NTDs and to better define how much disease is attributable to each pathogen.

Where concurrent infections occur, it will be important to determine whether infection with *any* NTD is sufficient (alone) for morbidity development, or if multiple infections have additive or synergistic (multiplicative) effects. Population-based studies are in progress to measure these interaction effects. In the meantime, it would be more appropriate to consider a combined NTD or a “polyparasitism” category as an operational diagnosis for disease burden rankings, rather than leave anemia, infertility, etc., as separate “diagnoses” in the GBD tables.

### Doctrinal views of the asymmetric effects of poverty on disease

*“Poverty is a lot like childbirth—you know it is going to hurt before it happens, but you'll never know how much until you experience it.”* J. K. Rowling, 2002 [42]. This quotation from an author who was once plunged into poverty, and later became one of the wealthiest women in the UK, highlights the very important differences between the theoretical contemplation of poverty and the actual experience of living with poverty [17].

Although in epidemiologic studies, location and environment have long been known to be important effect modifiers in disease formation, the GBD intentionally excluded patient context as a factor in the calculation of disease burden [11]. This decision was seen as necessary to create an interchangeable (fungible) unit of disease burden (i.e., the DALY) that could apply to all areas of the world, and could be used to “fairly” rank diseases by their “average” health burden [9]. The GBD framers rationalized their decision to avoid disease-context considerations on the basis of a controversial “like-as-like” moral argument [9]. The crux of the argument was that any disease should be seen as having the same impact on individual performance no matter where the disease occurs. Furthermore, any weighting or location-specific adjustment of disease burden was felt to lead to unwarranted exceptionalization of individual conditions, directly or indirectly contributing to a “welfarist” bias in disease burden assessments and indirectly contributing to bias in health policy prioritization [9]. However, as previously discussed, where significant population stratification occurs, as with wealth disparity, the heterogeneity of groups means that there are, in fact, two or more populations to evaluate, and there can be no “average” health burden impact.

Taking the GBD formulation of the DALY construct in its own historical context, one can infer that the individual-focused, antiwelfare agenda predominant in the US and UK government policies during the 1980s and 1990s may well have driven this decision to remove context from disease burden assessment. The free-market agendas popular at the time were based on laissez-faire economic theories that emphasize the importance of individual-level choices in determining success, while frequently denying a significant role of group-level factors, or “society,” in modifying life events. [43] Despite many counterarguments asserting that the contextualization of disease is imperative to the understanding of disease burden [11]–[13],[17], the environmental aspects of disability formation were excluded from the GBD system. In addition, although public health has long been viewed as a “common good” that requires collective or government-level decision-making [44], it is surprising that the strong influence of group-level factors in disease formation was not incorporated into the GBD assessments. The context of disease is particularly germane when we consider the well-recognized additive or multiplicative roles played by both individual-level and group-level poverty in determining many individual performance outcomes [45],[46].

With regard to context, the “like as like” argument of the GBD formulation is assumed to be *egalitarian,* in that burden of disease is identical for the same disease process for any individual, regardless of location or socioeconomic status. In practical terms, the “like as like” position is meant to ensure that the “currency” of disease burden is interchangeable, universally consistent, and comparable cross-culturally. DALY proponents argue that it is the best available system for assessing the global burden of disease and setting disease control priorities. Reidpath [12] asks, however, whether a paraplegic person in Niger has the same disability as a paraplegic person in Australia, where multiple support systems exist to facilitate personal function. The answer is quite clearly no, yet this glaringly obvious asymmetry is not addressed in the GBD system.

## Poverty-Related Challenges for the GBD DALY System

The presence of regional-, household-, and individual-level poverty has many important numerical effects on the estimation of DALY values for the NTDs.

A. The availability of adequate epidemiologic and vital statistics ­is highly inconsistent from country to country and from region to region, tending to be the most inaccurate where resources are most scarce [34]. Records of population size and mortality are incomplete, especially in regions with little infrastructure, where the NTDs are frequently most prevalent. Where hospitals and clinics are not accessible, accurate measures morbidity and mortality from NTDs can be difficult to obtain. For most sub-Saharan African countries, GBD burden has been extrapolated from the scant data available from other locations, meaning estimates will be only approximate (i.e., wrong), with a strong tendency towards underestimation of disease burden. In facing the context-specific effect of inadequate data availability, the GBD is probably overemphasizing the burden of diseases prominent in developed countries, where good epidemiological data are available, and undervaluing the burden of disease in developing countries.

B. The use of age-weighting factors in DALY calculations is highly controversial. Despite the egalitarian “like as like” stance of the GBD (see reference [6] for further detail), the weight attributed to morbidity or mortality is not equal across all human age groups [11]. Disease occurring among individuals in the range of 20–40 years of age is given the highest effective DW by this weighting, and disease among younger or older individuals counts for much less. The GBD's rationale is said to reflect “societal preference,” relating to an individual's productive capacity at different stages of life [9],[16]. Generally, it is assumed that the very young and the very old are dependent on the middle-aged for their care and subsistence; thus the loss of a year of healthy life in middle age is likely to have greater societal consequences [47].

However, the framework for age weighting does not take into consideration the highly significant cross-cultural variation in productive economic and social roles, and is biased toward a model of life stages only as they are known in developed economies [17],[48]. It is typical in non-Western cultures that children contribute substantially to household productivity as early as 4 years of age via child care and food production. For example, Mikea children of southwestern Madagascar contribute nearly enough foraged goods to the household to support their own caloric intake [49]. Similarly, in many cultures, the elderly do not retire or become dependent on younger workers within their society, but remain productive contributors to society [48]. Thus, in a developing economy where cash income is scarce, work starts at age 4, and life expectancy is 46 years, then the age-weighting system of the DALY calculation would clearly not reflect the “societal preference” for the value of any life years lived or gained by health interventions.

**Figure 2:**
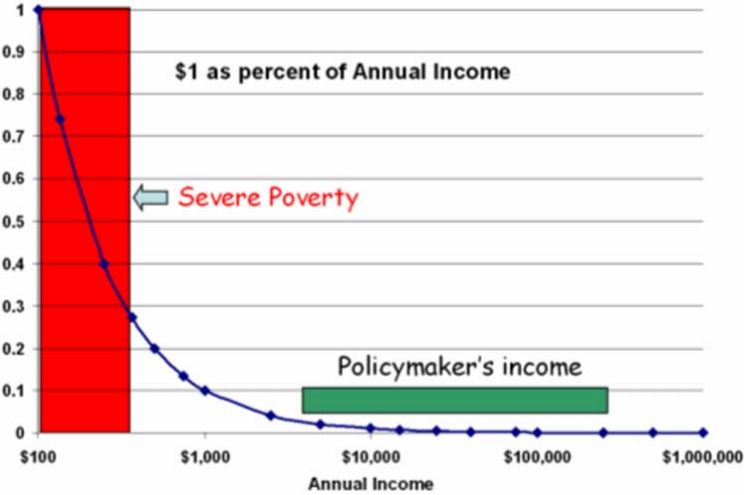
“Asymmetrical” or Nonlinear Outlook on Program Costs and Health Gains. To someone in the setting of severe poverty (income < $1 per day), the gain or loss of a single health or performance-related dollar will appear substantially more important than it will to a middle-income policymaker.

C. Not all morbidity comes to clinical attention, especially where health-care resources are limited. In the setting of poverty, the impact of physical impairments is proportionally larger, because in poor rural settings, hard physical labor is the predominant mode of survival. Small losses in productivity are highly leveraged in terms of individual and household productivity (Figure 2), and are economically more important to subsistence farmers and herders than to workers in more developed economies. This again raises the question as to whether PTO focus groups can serve as adequate proxies in making health care decisions for persons with NTDs. It is an established economic principle that the value of an economic transaction is determined by the utility of the purchase to the consumer alone. If PTO committees do not understand the impact of chronic illness in the context of poverty, they should not be the ones valuing the impact of NTDs. If PTO committees are, in fact, representing the values of the donors (or other payors), then the DALY is no longer a positivist “value-free” metric, as claimed in the GBD program's description and goals [9].

The socioenvironmental structure in which an individual lives dictates the capacity to which the individual is able to be functional and productive with a given disease, and thus also dictates the burden ensuing from the inability to function with disease within society [12]. There are many misconceptions about the impact of NTDs on daily life; for example, schistosomiasis infection is significantly associated with subclinical morbidities such as caloric undernutrition, exercise intolerance, fatigue, diarrhea, and abdominal pain [26], all of which can be interrelated and contribute to loss of productivity [50]. The relationship between NTDs and loss of productivity is supported by other studies of helminth infections that have found infection to be associated with decreased physical function [51], decreased productivity [52], and decreased wage-earning capacity in later adulthood in developing countries [50]. Anemia does not have to be severe to be disabling. Likewise, mild “chronic disease” anemia is significant in terms of lowered endurance, income [53], and worsened birth outcomes [54]. Similarly, undernutrition does not have to be severe to be disabling [55].

**Figure 3:**
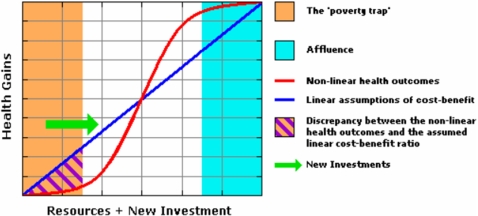
In the Context of Poverty There Are Nonlinear Differences in the Efficacy of Treatment on Health Outcomes. Individual poverty and residence in an impoverished environment can combine synergistically to impair improvement from single health interventions. (See reference [56].)

D. Due to nonlinear quantitative factors, common cost-effectiveness evaluations may fail to be relevant in the setting of the “poverty trap” [56]. In standard cost-effectiveness analysis (CEA), there is a tacit assumption of linearity between the size of health-care investment and the size of health benefits obtained (Figure 3) [57]. As DALYs are frequently used as outcomes or “utilities” in this type of exercise, a traditional CEA will prove invalid for NTDs for two reasons: first, because of misclassification of the nature and size of disease burden (DW), and second, because of the nonlinear effects of poverty on reversibility of disease burden.

Will an increment in investment for NTD control yield a predictable suite of DALY benefits for all treated populations? Probably not. Local variation in asymmetric poverty effects will vary program effects on health and performance, which means that expected number of “DALYs averted” [10] may or may not be achieved for a given investment (Figure 3). Because of the correlated confounding effects of local poverty [56], the rate of DALY improvement after program interventions may vary considerably between poor and middle-income locales.

E. Finally, alternative patient preference approaches (time trade-off, willingness-to-pay) will not work unless the alternative health states are truly understood. Patients with NTDs will not be well-informed consumers (Box 3) if they are not aware of the alternative health states to be considered. Wherever chronic NTDs are part of the fabric of daily life and people carry infection and its late consequences for most of their lives, can they appreciate disease impact without experiencing the difference? Can they be truly informed consumers? Here, education level and understanding of disease causation can play a highly significant role. In particular, one can see that willingness-to-pay and time trade-off exercises for disability ranking are likely to be invalid in mostly noncash economies and in settings where limited lifespan (i.e., less than 50 years) is usual. Results for these setting may be significantly different than for developed economies.

## Overview and Summary

*“If our knowledge is poor, a mathematical model will poorly reproduce reality – this is actually a useful implication because it can verify whether our knowledge about the infection is sufficiently complete.” —* Duerr, Dietz, and Eichner 2005 [58].

Overall, we can see that there is no “average disability” for many NTDs, but because just such a value is used in the complex DALY exercise [9], any current GBD comparisons reflecting on the “priority” of certain diseases over others [2] remain extremely dubious. In previous sections, we have identified a number of critical choices that result in inherent deficiencies in the current DALY valuations, particularly for the diseases of developing countries now referred to as the NTDs. Because the DALY calculations are the underpinnings of the GBD program's disease rankings, we conjecture that these flaws systematically undervalue priorities for controlling the diseases of the poor.

As the NTDs are given low priority, the net result of such “merely technical” DALY errors is to enshrine chronic NTD transmission and infection as the status quo, creating a situation in which chronic diseases and their related disabilities become an expected part of life [59],[60]. As a result, NTD-affected communities are less economically productive, and impoverished communities are less and less likely to break out of the “poverty trap,” a vicious cycle of negative economic growth related to individual and group-level poverty effects [56]. Achieving the poverty reduction objectives of the Millennium Development Goals will require that serious attention be paid to the far-reaching effects of NTDs—these neglected diseases are transmitted as a result of poverty and, because of their chronic disabling effects, remain a continuing cause of poverty.

Gwatkin and colleagues [18] point out the fact that although the GBD program suggests a reordering of priorities, stressing that noncommunicable diseases of older age groups are becoming more prevalent than communicable diseases [2], this supposed shift in prevalence does not reflect the reality of health issues in most poor areas. If health investments favor noncommunicable disease control (based on GBD DALY rankings), it turns out that rich areas stand to benefit much more from this investment (an increase of 5.3 years of life expectancy) than do poor countries (1.4 years). In Gwatkin's revised analysis of World Bank and GBD data, an investment that instead reduces communicable diseases would gain an additional 4 years of life expectancy for the world's poorest 20%. The substantial difference in life expectancy outcomes shows that it is still a priority to invest in control of communicable diseases, including the NTDs.

## Future Directions and Recommendations

Ultimately, we see that the GBD's “like as like” philosophy is *not* fair to those living in poverty. In particular, the approach to disability weighting used in the current GBD DALY system fails to accurately measure the health burden of NTDs. Can we repair the DALY to make it more accurately reflect the burden of disease in developing countries, or should we simply replace the DALY with a better health metric?

In 1996, the DALY approach was seen as a useful “first approximation” for mapping disease burden, both worldwide and across regions or countries. Its obvious limitations, particularly regarding DW determinations, make its current use in cost-effectiveness estimations outdated and unreliable. For the new GBD 2005 initiative, we should not accept that the original DALY DW estimates [6] reflect the world's “societal view” of NTD burden [17], nor should we use DALY rankings to define the “importance” of any disease. Those 1996 DALY estimates clearly reflect the cultural viewpoints of those who constructed the DALY, and not those of other cultures or of the NTD-endemic populations. If the disease control world insists on using a DALY-type metric for their comparisons, then a substantial revision, particularly with adjustment for disease and disability context [11], is essential. At the least, a weighted adjustment for individual or local poverty factors would be appropriate. If the DALY system remains in use, and it is to provide a valid measurement tool, then its GBD 2005 revision must include system-wide changes in the GBD calculations that address the concerns raised in this paper.

We believe that it would be better to use formal patient-based determination of quality-of-life (QoL) and quality-adjusted life-year (QALY [57]) for determination of losses due to NTD diagnoses in endemic locations, as has been recently done for chronic schistosomiasis japonica in China [61] and schistosomiasis mansoni in Kenya [62]. QALY values are estimated from preference-based health-related QoL interviews administered to groups of patients or to members of the general population [21]. QoL-related visual analog scales [63] and the short EuroQol 5D questionnaire [61],[64] have already been adapted for use in Africa and China. Determining QALY impacts for NTDs would take extensive effort in terms of fieldwork, but putting all things into perspective, it has been 20 years since the first DALY program was developed, and many of its systemic flaws (that we and others [11]–[13],[16],[26],[47],[65] have identified) have not been addressed since then. Use of a patient-based QoL assessment such as the QALY would get beyond the narrow, Northern (or developed-economy) slant in the disease burden assessments used in current versions of the GBD program, and provide a more realistic idea of the impact of NTDs on world health.

The recommended approach to restructuring the DALY should go well beyond efforts to improve incidence and prevalence data. While we applaud those technical corrections, the following are the minimal changes that are needed.

A. The DALY weights should be made internally consistent—for example, the impact of infertility should not vary whether it is postsepsis or due to sexually transmitted diseases [6].

B. Age weighting should not be used to value heath outcomes [11].

C. Clinical syndromes such as anemia and malnutrition are not diagnoses, and should not be confused with preventable causative etiologies. Where overlap occurs in terms of causation for such disabling syndromes, we need to recapture the attributable burden associated with all potentially preventable causes, including NTDs [26].

D. For chronic disabling conditions, DWs should be evidence-based, reflecting already available data on the physical and personal performance impacts of these diseases [26]. Scenarios for DW weighting of NTDs should be vetted both by patients and by disease specialists who know these diseases. GBD program facilitators should not assume that *their own* knowledge of a disease reflects the world's view of a disease's burden, nor should an affluent PTO panel assume that they encompass a patient's perspective on disease burden in the context of poverty.

E. DWs should be adjusted for health resource abundance in the location/context where the disease occurs [11].

As a first step, we recommend a revaluation of disease-specific DWs based on available evidence and conditional prevalence of disease-related morbidities that occur with NTD infections [26],[65],[66]. It will be important to include the very important domains of physical productivity and the culture-dependent, stigma-causing disease outcomes such as infertility, low fecundity, loss of marriage prospects, and loss of employability that were clearly not addressed in the initial GBD assessments [14],[66].

Where attributable risk for identified morbidities cannot be disaggregated, an attempt should be made to examine the joint NTD causation of syndromic disabilities, especially anemia and undernutrition. These two important NTD-related outcomes cannot be left as catchall “diagnoses” if the benefits of available drug-treatment and transmission-prevention measures are to be fairly assessed. Given that many NTD control programs are now providing multiple-drug administration with agents that provide treatment of several NTD infections simultaneously [8],[67], it would be appropriate to evaluate the combined burden of a common “basket” of NTD infections such as hookworm disease, ascariasis, and lymphatic filariasis as an operational NTD disease construct, which then becomes the appropriate analytic level for cost-effectiveness [68].

Overall, there needs to be a regular reassessment of disease burden as local developmental conditions change. This should go well beyond just updating population and prevalence data. We favor the use of QALYs as more comprehensive, “societal” view of disease impact, particularly one that captures the disease externalities related to poverty that were not appreciated or captured by the standard DALY valuation approaches [57],[61]. It is hoped that the GBD 2005 project will pay heed to these issues. Otherwise, the new, “second-generation” DALY system of the new GBD 2005 will be as deficient as the first Global Burden of Disease program in assessing NTD-related health burden.
